# Brown fat triglyceride content is associated with cardiovascular risk markers in adults from a tropical region

**DOI:** 10.3389/fendo.2022.919588

**Published:** 2022-07-19

**Authors:** Milena Monfort-Pires, Giulianna Regeni-Silva, Prince Dadson, Guilherme A. Nogueira, Mueez U-Din, Sandra R. G. Ferreira, Marcelo Tatit Sapienza, Kirsi A. Virtanen, Licio A. Velloso

**Affiliations:** ^1^ Laboratory of Cell Signaling, Obesity and Comorbidities Research Center, State University ofCampinas (UNICAMP), Campinas, Brazil; ^2^ Department of Nutrition, School of Public Health -University of São Paulo, São Paulo, Brazil; ^3^ Turku PET Centre, University of Turku, Turku, Finland; ^4^ Turku PET Centre, Turku University Hospital, Turku, Finland; ^5^ Department of Epidemiology, School of Public Health-University of São Paulo, São Paulo, Brazil; ^6^ Division of Nuclear Medicine, Department of Radiology and Oncology, Medical School of University of São Paulo (FMUSP), São Paulo, Finland; ^7^ Clinical Nutrition, Institute of Public Health and Clinical Nutrition, University of Eastern Finland (UEF), Kuopio, Finland

**Keywords:** thermogenesis, ^18^F-fluorodeoxyglucose (^18^F-FDG), positron - emission tomography, magnetic resonance imaging (MRI), fat, inflammation, dyslipidemia

## Abstract

Brown adipose tissue (BAT) is regarded as an interesting potential target for the treatment of obesity, diabetes, and cardiovascular diseases, and the detailed characterization of its structural and functional phenotype could enable an advance in these fields. Most studies evaluating BAT structure and function were performed in temperate climate regions, and we are yet to know how these findings apply to the 40% of the world’s population living in tropical areas. Here, we used ^18^F-fluorodeoxyglucose positron emission tomography – magnetic resonance imaging to evaluate BAT in 45 lean, overweight, and obese volunteers living in a tropical area in Southeast Brazil. We aimed at investigating the associations between BAT activity, volume, metabolic activity, and BAT content of triglycerides with adiposity and cardiovascular risk markers in a sample of adults living in a tropical area and we showed that BAT glucose uptake is not correlated with leanness; instead, BAT triglyceride content is correlated with visceral adiposity and markers of cardiovascular risk. This study expands knowledge regarding the structure and function of BAT in people living in tropical areas. In addition, we provide evidence that BAT triglyceride content could be an interesting marker of cardiovascular risk.

## Introduction

Obesity is one of the most important risk factors for cardiovascular disease, and the worldwide increase in the prevalence of obesity has had an undisputed epidemiological impact on the current alarming rates of cardiovascular events (1, 2). There are multiple mechanisms linking obesity to cardiovascular diseases, such as subclinical systemic inflammation, insulin resistance, diabetes, dyslipidemia, and hypertension (3). However, it is expected that other, still-unknown factors, could play important roles in this scenario. Recent studies have suggested that the activity of the brown adipose tissue (BAT) could be related to cardiovascular conditions (4–8). A retrospective study evaluating more than 52,000 patients submitted to ^18^F-fluorodeoxyglucose positron emission tomography-computed tomography scans (^18^F-FDG PET/CT) revealed that BAT glucose uptake was inversely correlated with cardiovascular disease, which was independent of diabetes, hypertension, and dyslipidemia (7). Thus, further studies are needed to advance our understanding of the putative mechanisms linking BAT and cardiovascular abnormalities.

Most clinical studies evaluating BAT employ ^18^F-FDG PET/CT to determine glucose uptake as a surrogate marker of BAT activity. However, there is evidence suggesting that the distinct methodological protocols used by several groups could negatively impact the homogeneity and interpretability of data collected worldwide (9, 10). One of the problems is based on the use of static image acquisition, which limits the determination of glucose uptake, a dynamic process, to a single moment (11–15). Imaging, using CT scans, could also be improved; CT scans expose patients to extra radiation and provide images of lower soft-tissue definition as compared to magnetic resonance. In this context, the use of magnetic resonance imaging (MRI) as an alternative for the detection of non-activated BAT, independent of cold exposure and glucose metabolism, has been proposed (12–15) and tested (13, 16–19). The results suggest that this method reduces interexperimental variability (13, 16–18). In addition, they show that the content of BAT triglycerides (TG) is associated with insulin sensitivity and systemic lipid levels (18). However, it is unknown whether BAT TG is somewhat related to markers of cardiovascular fitness.

Here, we investigated the associations between BAT activity, volume, metabolic activity, and BAT content of triglycerides with adiposity and cardiovascular risk markers in a sample of adults living in a tropical area. We used ^18^F-FDG PET/MRI to determine the associations between BAT activity (mean standardized uptake value - SUV), volume (mL), metabolic activity (SUV*mL), and BAT TG content with body composition, lipid profile, and markers of inflammation in lean volunteers and volunteers with overweight or obesity. The study was performed with volunteers living in a tropical area and adds important information regarding a group of people that has been understudied regarding BAT activity (20–23).

## Methods

This study was a secondary analysis of the baseline measurements of a 4-week clinical trial that investigated the effects of olive oil on BAT activation (21) (Clinical trial registry: clinicalcrials.gov NCT03024359).

### Study subjects

The study was conducted from April 2017 to May 2019. Before the beginning of the study, all volunteers read and signed a written informed consent and the study was approved by three ethics committees at the institutions where the data were collected: Faculty of Medical Science of the University of Campinas (CAAE 60698716.1.0000.5404), Faculty of Medicine of the University of São Paulo (CAAE 60698716.1.0000.5404), and the School of Public Health of the University of São Paulo (CAAE: 60698716.1.3002.5421). All volunteers were recruited using electronic adverts on social media. Initially, 231 volunteers were screened and 109 fulfilled the eligibility criteria. Due to the complexity and duration of the protocol (about 4 hours), many volunteers were unable to join it. From 109 volunteers that fulfilled the criteria, 49 were included in the study; four were excluded due to incomplete images in PET/MRI; thus 45 volunteers completed the study (**Supplementary Figure S1**) (24). The inclusion criteria were men and women, 25- to 40-years-old, with BMI of 18.50 to 34.99 kg/m^2^. The exclusion criteria were the individual olive oil intake greater than 250 ml/month; ongoing pregnancy; diagnosis of severe neurologic or psychiatric diseases; use of anti-obesity or hypolipidemic medication; use of adrenergic or benzodiazepine drugs; diagnosis of cancer, communicable diseases, rheumatic diseases, liver or kidney failure, thyroid dysfunction or diabetes mellitus; +5% change in body mass during the previous six months; on any dietary approach aimed at body mass modification; metallic prosthesis. As this is an exploratory study, we calculated the sample size based on the capacity to determine BAT activity, which is the only data available for such calculation. According to Becher (7) the evaluation of 134.000+ PET-CT scans revealed that 5.9% had detectable BAT. Therefore, to calculate sample size, we employed the following parameters: confidence interval, 95%; margin of error, 7%; the proportion of the population with detectable BAT, 6%. This calculation resulted in a sample size of 45 individuals. Thereafter, considering that we had enough patients to detect variability in BAT, we asked if parameters related to BAT, such as SUV, BAT volume, BAT TG content, or BAT SUV max, would be associated with markers of CVD. Moreover, considering these comparisons have never been performed in previous studies, we could not calculate the sample size for each of the analyzed parameters.

### Study design

**Figure 1:**
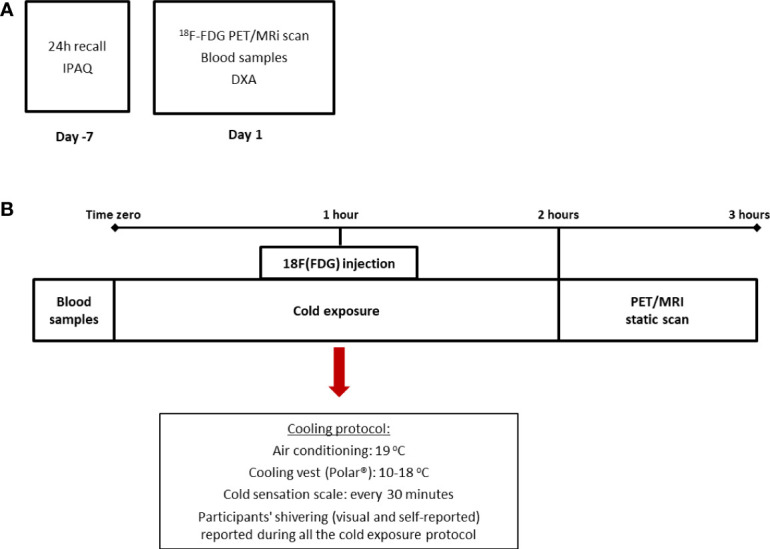
Design of the study and cooling protocol. The cross-sectional design of the study in which dietary and physical activity data were collected prior to the beginning of the PET/MRI scan **(A)** and cooling protocol **(B)** in which subjects were exposed to two hours of mild cold (air conditioning and cooling vest). IPAQ: International Physical Activity Questionnaire.

This study is a cross-sectional analysis of the baseline measurements of a 4-week clinical trial (21). For the purposes of this study, all subjects underwent one ^18^F-FDG PET/MRI for brown adipose tissue quantification and a DXA scan for body composition. In addition, data regarding anthropometry, diet, and physical activity were collected, as well as blood samples for several determinations (**Figures 1A, B**).

### Clinical variables

All clinical data were collected by trained staff. Height was measured using a fixed stadiometer and weight was recorded when individuals were wearing light clothing and no footwear, in a standing position on a Filizola digital scale. BMI was calculated as weight in kilograms divided by height in meters squared and waist circumference was measured at the midpoint between the bottom of the rib cage and the top of the iliac crest. Blood pressure was measured at rest in a sitting position using an oscillometer automatic device (Omron HEM-712C, Omron Health Care, USA). Body composition was assessed by dual-energy X-ray absorptiometry (DXA Lunar, General Electric^®^). For the purposes of this study, the following measurements were recorded: total and relative fat mass, total lean mass, and visceral fat mass, android/gynoid ratio, arm fat (in kg and %), leg fat (in kg and %) and trunk fat (in kg and %). For the L3-L4 visceral fat mass calculation, the fat fraction images of the MRI were used, and analyses were performed using CARIMAS software version 2.9 (Turku PET Centre). To determine the content of visceral fat, a volume of interest (VOI) in the abdominal area between the two vertebrae (L3-L4) was drawn, and all tissue with less than 40% triglyceride content was excluded. A visual slice-by-slice check to determine possible discordant areas was performed by the primary investigator. After excluding other tissues and subcutaneous adipose tissues, the remaining area was regarded as visceral fat (**Supplementary Figure S2**). For subcutaneous adipose tissue, the same abdominal area (between L3 and L4) and the same fat-content threshold (40%) were used. An example of how the assessment of visceral adipose tissue was carried out is depicted in **Supplementary Figure S2**.

### ^18^F-FDG PET/MRI scanning protocol

To promote the activation of BAT, we used an acute and individualized cold exposure protocol. Briefly, subjects were placed seated in an air-conditioned room (at 19°C) with a cooling vest (Polar Products^®^, Stow, OH) at 14–18°C for 2 hours. To avoid shivering, a visual scale of cold sensation was employed every 30 minutes (**Figure 1B**). Moreover, one of the researchers was responsible for checking visual shivering every 15 minutes. Both the air conditioning and the cooling vest temperatures were adjusted in case participants reported beginning of shivering. After 1 hour of cold exposure, a bolus of 185 MBq of ^18^F-FDG was injected and the participants remained for 1 hour under cold exposure before PET/MRI analysis. For the static PET/MRI scan volunteers were placed supine in a headfirst position inside the PET/MRI scanner (General Electric^®^). The MRI data were also collected after cold exposure. Thirty-six out of 45 volunteers included in this study followed this protocol, whereas nine volunteers were submitted to a 2-hour cold exposure, followed by the ^18^F-FDG injection inside the PET/MRI machine and collection of both dynamic and static data. For this study, only the static data (in SUVs) was considered and there were no differences in BAT activation between the two groups (data not shown).

### [^18^F] FDG PET/MRI data analysis

PET/MRI images were analyzed using CARIMAS 2.9 software (Turku PET Centre, Turku, Finland). For BAT activity the mean standardized uptake values (mean SUVs) were estimated by drawing a volume of interest (VOI) with the *mask* tool of the software on the fused ^18^F-FDG PET and MR images (as an anatomical reference). The VOIs were drawn on both sides (left and right) in the cervical and supraclavicular areas and the mean value for the two sides, adjusted to body weight, was determined. In addition, the BAT maximum SUV (SUV max) was determined as the highest SUV value among the voxels drawn in the VOI. For the estimation of BAT volume, we used the fused PET/MRI images in the cervicothoracic region (including cervical, supraclavicular, and axillary adipose tissues). The VOIs of potential BAT sites were drawn manually from the chin to the armpit and a threshold of 40% of triglyceride content was used in the fat fraction MR images. After the first threshold, the VOI was masked to the background (PET) and the second threshold of voxels with SUVs higher than 1.5 was used. Only tissues with a high TG content (>40%) and with high SUV (>1.5) were considered as active BAT for volume calculations. For BAT metabolic activity, the mean SUV was multiplied by the BAT volume (SUV*mL). Subcutaneous white adipose tissue activity was calculated as the mean SUV of a VOI drawn in the subcutaneous adipose tissue on the right side of the umbilical area. The content of triglycerides was calculated in the same region using the fat fraction maps. To avoid marking other tissues as BAT, the VOIs were not drawn close to the muscles and the threshold of 40% of TG was always employed. Additionally, the first author analyzed the same image on two different occasions to confirm SUV, volume, and content of TG values for each participant; no significant differences between measurements were observed.

### Biochemical determinations

Eight-hour fasting venous blood samples were collected. All subjects were instructed to abstain from alcohol and to avoid moderate to intense physical activity 48 hours prior to blood and ^18^F-FDG PET/MRI data collection. Blood samples were centrifuged, and plasma and serum were frozen at -80°C for further determinations. Fasting glucose was assessed using an Accu-Chek blood glucose monitoring system (Roche). Enzymatic colorimetric methods were used to determine triglycerides, total cholesterol, and HDL-c concentrations while LDL-c was obtained using the Friedewald equation. Interleukin 6 (assay range 3.1 - 300 pg/mL, sensitivity 0.7 pg/mL), monocyte chemoattractant protein-1 (assay range 31.2 - 2,000 pg/mL, sensitivity 10 pg/mL), insulin (15.6 - 500 pmol/L, sensitivity 2.15 pmol/L), and FGF-21 (assay range 31.3 - 2,000 pg/mL, sensitivity 8.69 pg/mL) were measured by enzyme-linked immunosorbent assay (ELISA) (R&D Systems, Minneapolis, MN, USA) as well as CXCL14 (assay range 125.0 - 4,000 pg/mL) (RayBiotech, Peachtree Corners, GA, USA). Insulin resistance (IR) was estimated by HOMA-IR (25).

### Dietary and physical activity data

Dietary data were collected prior to the beginning of the study (one week or less). Trained staff collected two 24-h recalls on non-consecutive days. The calculation of dietary intake was performed using NDSR (Nutrition Data System for Research, University of Minnesota, US). Physical activity data were collected using the International Physical Activity Questionnaire (IPAQ) (26); total and leisure-time physical activities were calculated in minutes per week while total seating time was estimated in hours per week.

### Statistical analysis

Statistical analyses were performed using SPSS version 22.0 and GraphPad Prism 8.0. All values are presented as mean and standard deviation. The Kolmogorov-Smirnov test was used to check the normal distribution of variables. For non-parametric variables, the equivalent non-parametric test was used. For comparisons between lean and participants with overweight or obesity, an independent t-test, or Mann-Whitney (for variables without normal distribution) was used. Spearman’s rank correlation was performed to analyze associations between brown adipose tissue with body adiposity and biochemical markers. In addition, logistic regression models were employed to investigate the associations between different body composition parameters and BAT activity (mean SUV), BAT volume, and the TG content of BAT. Because we observed associations between BAT TG and CV risk markers, we performed an analysis of variance (ANOVA) to detect differences among groups and assess how the groups differ and by how much. The ANOVA was employed to test differences in BAT activity, volume, and TG content across tertiles of total fat mass and visceral fat mass. A p-value of less than 0.05 was considered statistically significant.

## Results

Out of the 45 volunteers included in this study, 30 (66%) were lean (BMI<25 kg/m^2^), 5 (11%) were overweight (BMI ≥25<30 kg/m^2^) and 10 (22%) had obesity (BMI≥ 30 kg/m^2^). For this study, individuals with overweight and obesity were grouped and compared against lean. Sociodemographic data indicate that the subjects in the two groups did not differ regarding race/ethnicity (70.0 versus 66.7% Caucasian, for lean and participants with overweight/obesity, respectively) or level of education attainment (83.3 versus 86.6% finished college, for lean and participants with overweight/obesity, respectively). Both groups were also similar regarding the family record of obesity (67.0 versus 53.3% with no family record, lean and participants with overweight/obesity, respectively), diabetes (63.0 versus 60.0% with no family record, lean and participants with overweight/obesity, respectively), hypertension (45.0 versus 50.0% with no family record, lean and participants with overweight/obesity, respectively) and stroke and myocardial infarction (less than 15% family record for both groups).

Data regarding body composition, BAT measurements, blood biochemistry, and blood hormone levels are shown in **Table 1**. The female/male ratio (p=0.75, chi-square test) and the mean age (p = 0.22, Student’s t-test) were similar in the two groups. As expected, the two groups differed regarding all body composition parameters participants with overweight/obesity had a higher BMI, waist circumference, total fat mass, visceral fat mass, and total lean mass (p<0.01 for all parameters). However, in contrast with data published elsewhere (27–29), in the present cohort, there were no differences in BAT activity (mean SUV), BAT volume (mL), or BAT metabolic activity (mean SUV*mL) between groups (p = 0.89, p = 0.42, and p = 0.49, respectively). The BAT TG content was the only difference observed between groups, and this was greater in the participants with overweight/obesity, as previously reported (13, 16, 18). Lean volunteers presented higher glucose uptake in the subcutaneous white adipose tissue, whereas subjects with overweight/obesity had a higher TG content (p<0.01). In addition, lean volunteers had lower fasting glucose, LDL-c, and triglycerides, and higher HDL-c. HOMA-IR was also lower in this group. For both total cholesterol and systolic blood pressure, the differences between groups reached borderline significance (p = 0.06 for both), and both were higher in the volunteers with overweight/obesity. Regarding the circulating markers of BAT activity, CXCL14, secretin, and 12,13-dihydroxy-9Z-octadecenoic acid (12,13 di-HOME) were similar in the two groups, whereas there was a trend for higher FGF21 in the subjects overweight/obesity. **Supplementary Figure S3** shows data regarding temperature and cold sensation (24). Mean outside temperature in the one-month period prior to the study (**Supplementary Figure S3A**) (24), mean vest temperature during cold exposure (**Supplementary Figure 3B**) (24), and mean cold sensation (**Supplementary Figure S3C**) (24) were similar for lean subject and volunteers overweight/obesity. Dietary and physical activity data are shown in **Supplementary Table S1** (24). Lean volunteers presented a trend for more physical activity than subjects with overweight/obesity (p = 0.06); nevertheless, there were no differences in sedentary behavior or dietary intake between groups. The correlations of parameters related to BAT are shown in **Supplementary** (24). Because the volume was calculated using a threshold of 1.5 SUV and the SUV max represents the highest SUV in the supraclavicular area, BAT activity, SUV max, volume, and metabolic activity were highly correlated among each other (p<0.05, **Supplementary Table S2**). On the other hand, the TG content of BAT was not correlated with any of the other variables, which was, somehow, unexpected as inverse correlations between BAT activity and content of TG have been reported (30).

**Table 1:** Sex, age, body composition, brown adipose tissue, white adipose tissue, clinical data, blood biochemistry, blood hormones and inflammatory markers in the volunteers.

	Lean(n=30)	Overweight/obesity (n=15)	p between groups	Total sample (n=45)
**Women (%)**	60.0	53.3	0.75	58%
**Age (years)**	31.9 ± 4.8	33.8 ± 5.3	0.22	32.51 ± 4.97
***Body composition***				
**BMI (kg/m^2^)^¥^**	21.8 ± 1.9	31.1 ± 2.9	<0.01	24.9 ± 5.0
**Waist circumference (cm)**	77.1 ± 7.4	98.4 ± 5.4	<0.01	84.2 ± 12.2
**Total fat mass (kg)^¥^**	17.0 ± 3.1	32.6 ± 7.7	<0.01	22.2 ± 9.0
**Total fat mass (%)**	27.8 ± 5	37.7 ± 8.2	<0.01	31.1 ± 7.8
**Visceral fat mass (g)^¥^**	218.2 ± 192	1093.5 ± 502.3	<0.01	510.0 ± 527.9
**L3-L4 visceral fat mass (g)^¥^**	33.1 ± 25.4	110.9 ± 41.1	<0.01	59.1 ± 48.4
**Total lean mass (kg)**	42.5 ± 8.7	51.5 ± 10.6	<0.01	45.5 ± 10.2
***Brown adipose tissue***				
**BAT activity (mean SUV)^¥^**	0.9 ± 1.0	0.8 ± 0.9	0.89	0.9 ± 1.0
**BAT volume (mL)^¥^**	8.5 ± 17.2	18.3 ± 37.7	0.42	11.4 ± 25.7
**BAT content of triglycerides (%)^$^**	69.4 ± 5.5	77.7 ± 5.1	<0.02	72.6 ± 6.7
BAT met. activity (mean SUV*ml)^¥^	24.4 ± 80.7	38.6 ± 80.2	0.49	28.1 ± 78.2
**White adipose tissue**				
**Sub. WAT activity (mean SUV)**	0.26 ± 0.02	0.16 ± 0.02	<0.01	0.23 ± 0.1
**Sub. WAT content of TG (%)**	79.9 ± 1.8	90.0 ± 1.0	<0.01	83.3 ± 9.5
***Clinical data***				
**Systolic blood pressure (mmHg)^#^**	110.6 ± 13.5	119.9 ± 10.5	0.06	113.7 ± 13.2
**Diastolic blood pressure (mmHg)^#^**	67.9 ± 10.2	70 ± 13.4	0.62	68.6 ± 11.2
**Fasting glucose (mg/dL)**	75.6 ± 9.8	81.5 ± 7.6	0.046	77.6± 9.4
**HOMA-IR^¥^**	0.7 ± 0.2	1.3 ± 0.6	<0.01	0.9 ± 0.5
**Total cholesterol (mg/dL)**	185.2 ± 28.8	204.4 ± 33.7	0.06	191.4 ± 31.4
**LDL cholesterol (mg/dL)**	110.0 ± 23.3	129.5 ± 28.6	0.02	116.3 ± 26.4
**HDL cholesterol (mg/dL)**	55.0 ± 9.8	46.1 ± 11.7	0.01	52.1 ± 11.2
**Triglycerides (mg/dL)^¥^**	78.2 ± 38.7	124.5 ± 68.8	<0.01	93.3 ± 54.3
**Castelli I index**	3.4 ± 0.7	4.5 ± 1.3	<0.01	3.9 ± 1.1
**Castelli II index**	2.1 ± 0.6	2.9 ± 0.9	<0.01	2.4 ± 0.8
**CXCL14 (pg/mL)^¥^**	4893.7 ± 3793.2	5103.0 ± 3643.4	0.27	4956.5 ± 3703.4
**FGF21 (pg/mL)^¥^**	76.8 ± 56.1	112.9 ± 72.3	0.06	88.5 ± 63.2
**Secretin (pg/mL)^$^**	403.7 ± 64.4	382.1 ± 50.8	0.41	396.4 ± 60.3
**12,13 Di-HOME (ng/mL) (n=20)**	17.5 ± 6.3	14.1 ± 6.5	0.21	16.2 ± 6.4

¥ non-parametric t test/# n=33/$ n = 39.

**Figure 2:**
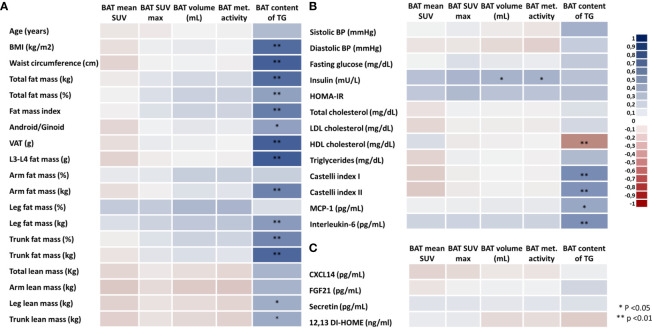
Heatmap of correlations between brown adipose tissue parameters, body adiposity and blood biomarkers. BAT activity (mean SUV), BAT maximum SUV, BAT volume (mL), BAT metabolic activity (SUV*mL) and BAT content of triglycerides **(A)**, biomarkers **(B)**, and markers of BAT activity **(C)** in all volunteers. Different colors indicate the intensity of the correlation coefficient (from the darkest red with correlation coefficient of -1.0 to the darkest blue with correlation coefficient of 1.0).

To investigate the associations between BAT activity (mean SUV), BAT maximum SUV (SUV max), BAT volume (mL), BAT metabolic activity (mean SUV*volume), and BAT TG content (%) with body adiposity parameters, we performed Spearman’s correlation using BAT data from PET/MRI and measurements of body adiposity from DXA (**Figure 2**). Because the lean volunteers and the participants with overweight/obesity did not differ regarding BAT activity or volume, all subjects were included in the analysis. Contrary to what has been described for BAT activity and volume in other studies, in our sample there were no associations between body adiposity, waist circumference, or visceral fat mass and parameters related to BAT activity and size (**Figure 2A**). In contrast, there were strong and significant associations between the TG content of BAT with almost all body adiposity parameters. Among all the different fat distributions, only leg fat mass was not positively associated with the TG content of BAT. In addition, stronger associations were detected between the TG content of BAT and central adiposity (waist circumference, visceral adipose tissue, L3-L4 fat mass and trunk fat mass) while no correlations were detected between lean mass and BAT TG content. Furthermore, the TG content of BAT was directly associated with the blood lipid Castelli indexes, blood MCP1, and IL-6, and inversely associated with HDL-c (**Figure 2B**). Regarding other parameters, there was a positive correlation between BAT volume and BAT metabolic activity with blood insulin levels (**Figure 2B**). Additionally, we observed that adjusting to baseline physical activity level did not affect the correlation coefficient, so crude correlations are depicted in **Figure 2**. In a stratified analysis, most of the associations were lost for the lean group (**Supplementary Figure S4A**) (24), but the TG content remained significantly correlated with the L3-L4 fat mass and secretin (**Supplementary Figure S4A**) (24). Of note, in the lean group, both insulin and HOMA-IR were correlated with all markers of BAT activity (**Supplementary Figure S4B**), whereas systolic blood pressure was inversely associated with both volume and BAT metabolic activity. Conversely, for the group of volunteers with overweight/obesity (**Supplementary Figure S5**) (24), the associations between body adiposity (mostly central adiposity) and TG content of BAT were maintained. BMI, waist circumference, visceral fat mass, and trunk fat mass were all correlated with BAT TG content (**Supplementary Figure S5A**) (24), whereas no associations were detected for BAT activity or volume. In addition, BAT TG content was inversely correlated with HDL-c (**Supplementary Figure S5B**) (24) and IL-6, whilst the browning marker CXCL14 was negatively correlated with all markers of BAT activity.

**Figure 3:**
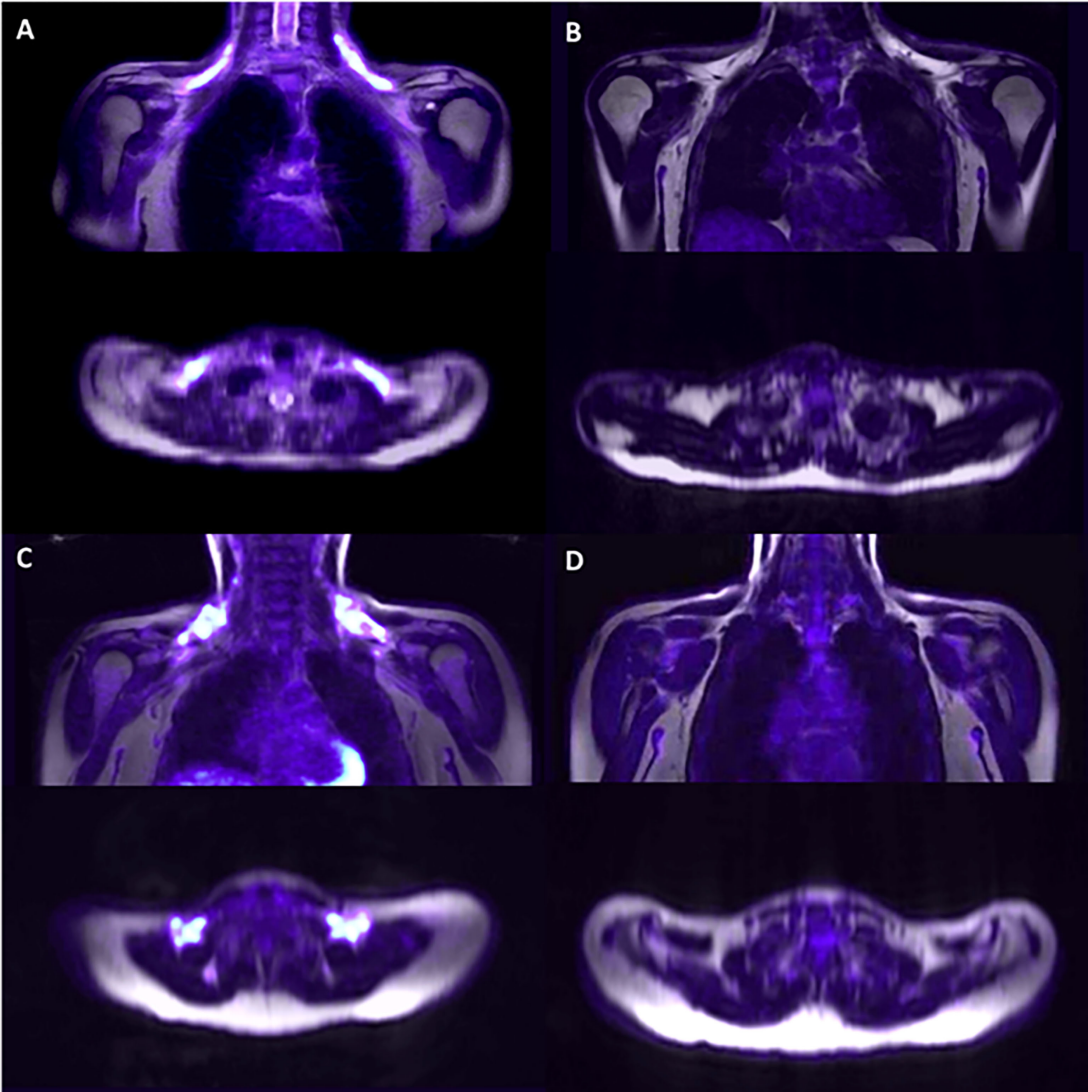
Representative PET/MRI of the supraclavicular area after cold exposure. Supraclavicular area of lean subjects with detectable BAT **(A)** and non-detectable BAT **(B)** and subjects with overweight/obesity with detectable BAT **(C)** and non-detectable BAT **(D)**.

Representative PET/MRI images obtained from volunteers with active and inactive BAT in both groups are depicted in **Figure 3**. Among participants, 30% had no detectable BAT volume/metabolic activity; and, contrary to what would be expected, the proportion of individuals with no BAT volume was slightly greater in the lean group as compared to the volunteers with overweight/obesity (36.6% versus 13.3%; p = 0.096).

To better investigate whether sex and age could influence the associations between adiposity and BAT activity, volume, and TG content, we performed uni- and multivariate logistic regressions using mean SUV (<1.2 or ≥1.2), BAT volume (no/yes), and TG content (<72% or ≥72%) [as reported before in (13, 16–18)] as dependent variables, and body composition parameters in either categories (BMI and waist circumference) or in tertiles (for all other variables). The results of the logistic regression are shown in **Supplementary Table S3**. In both uni- and multivariate logistic regression analyses, increases in body composition parameters led to a greater odds ratio for having high BAT TG content but did not affect BAT activity (mean SUV) or volume. In multivariate regression analysis, having a high BMI or waist circumference increased the odds ratio of having a high TG content by 35- and 27-fold, respectively (**Supplementary Table S3**). In addition, having a high total fat mass, visceral adipose tissue, trunk fat, L3-L4, or high Castelli I index was predictive of a higher TG content, increasing the odds ratio by 184.2-, 189.0-, 72.8-, 50.6- and 13.0-fold, respectively when comparing the third tertile against the reference. The opposite pattern was detected for HDL-c in which higher tertiles were associated with lower TG values (P<0.05). No differences across tertiles of body composition parameters were detected for BAT activity or BAT volume (p>0.05 for all analyses).

**Figure 4:**
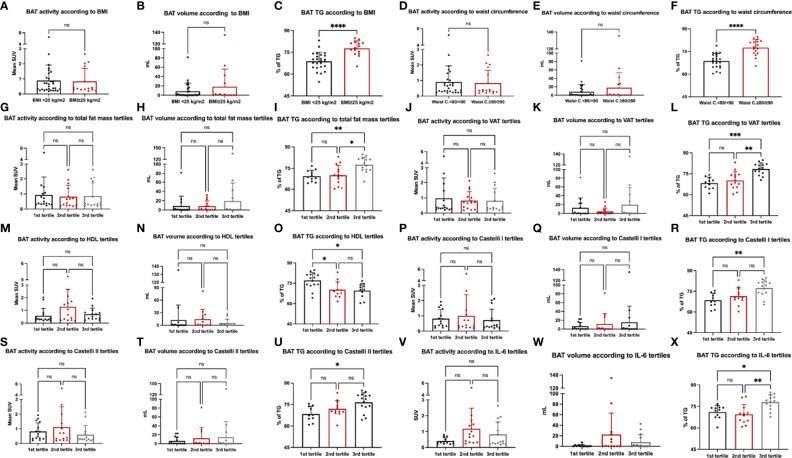
Markers of cardiovascular risk and BAT. Analysis of variance (ANOVA) was performed for BMI **(A–C)**, waist circumference **(D–F)**, total fat mass **(G–I)**, visceral adipose tissue **(J–L)**, HDL-c **(M–O)**, Castelli I **(P–R)** and Castelli II index **(S–U)** and IL-6 **(V–X)** with BAT activity, volume and BAT content of TG.

Because we did not find associations between BAT activity, volume, or metabolic activity with adiposity or markers of CVD risk, we sought to investigate whether categories of these markers could be associated with BAT in a dose-dependent manner. Using an analysis of variance, we aimed to detect differences among categories of adiposity and CV risk markers and assess how much these groups differ regarding BAT activity, volume, and content of TG. Therefore, we performed ANOVA comparing BMI, waist circumference, total fat mass, visceral adipose tissue, HDL-c, Castelli I and Castelli II indexes, IL-6 and MCP-1 against BAT activity, volume, and TG content (**Figure 4**). As for the analyses of correlations, BAT mean SUV and volume did not differ across categories of BMI (**Figures 4A, B**) or waist circumference (**Figures 4D, E**), or across tertiles of total fat mass and visceral adipose tissue (**Figures 4G, H, J, K**), HDL-c (**Figures 4M, N**), Castelli I and II indexes (**Figures 4P, Q, S, T**) or IL-6 (**Figures 4V, W**). Regarding the TG content, we detected a difference between low and high BMI and waist circumference (**Figures 4C, F**) and between the first and 3rd tertile of total fat mass and visceral fat mass (**Figures 4I, L**). In addition, differences between tertiles were observed for HDL-c (**Figure 4O**), Castelli I and Castelli II indexes (**Figures 4R, U**), and IL-6 (**Figure 4X**).

As the results herein reported are somewhat different from those reported elsewhere (27–29, 31), particularly regarding data collected from subjects living in temperate climate regions, we performed a review of the literature to determine whether the lack of association between BAT activity and adiposity and markers of cardiovascular risk could be related to differences in environmental temperature. Thus, we analyzed differences in BAT activity values in people from tropical and temperate climate countries (6, 18, 19, 32–43)(**Supplementary Table S4**) (24). Very few studies investigated BAT activity in tropical countries and most of them were done in Australia and Brazil (19–23, 44–50). Although the methods differed across studies, it was clear that studies conducted in tropical regions usually presented lower SUV values and could hinder associations between BAT activation and adiposity, for example. Moreover, dynamic scan data from our study (collected in a subsample of 8 volunteers) may suggest that the glucose uptake rate of glucose is also lower in Brazil (GUR: 0.94-1.0 µmol × min-1 × 100 g-1) (**Supplementary Table S4**) (23). Regarding the TG content, few studies analyzed PET/MRI data; however, for those that did, the values ranged from 70 to 90%, like the values reported in this study (13, 16, 18, 51).

## Discussion

In this study, we investigated the associations between BAT activity, volume, and content of triglycerides, with several markers of adiposity and cardiometabolic risk in a cohort of 45 volunteers living in a tropical country. Unlike most studies reporting data from people living in temperate climate areas (9, 27, 29, 31, 38, 52), in this cohort we found no associations between BAT activity or volume and adiposity. Nevertheless, we found strong positive correlations between TG content, adiposity, and markers of cardiovascular risk in both lean volunteers and participants with overweight/obesity.

The description of BAT in adult humans in 2009 (31, 38, 52) leveraged interest in the field, as BAT emerged as a potential target for the treatment of obesity, metabolic and cardiovascular diseases. Most early studies revealed an inverse association between BAT activity and whole-body adiposity (27–29, 31, 52, 53), although some studies found the converse (54). One of the reasons for this discrepancy seems to be the environmental temperature in which the analyzed volunteers live. However, as the number of studies undertaken in temperate climate regions outnumbers by far those performed in tropical areas, further studies are needed to provide consistency regarding this concept.

Currently, most studies employ cold-exposure stimulation as part of the strategy aimed at intensifying BAT signal in ^18^F-FDG PET/CT; under this condition, they report an inverse association between body adiposity and BAT activity (27, 28, 31, 37). However, this is not a rule; in a recent study, Spanish researchers showed that BAT volume was positively correlated with BMI, fat mass and percentage of fat mass in men, which conflicts with previous studies conducted in Europe (54). Moreover, they reported no association between BAT volume and adiposity in women. As in Brazil, the differences between the mean outdoor temperature in Spain, when compared to Finland, Canada, and the USA for example, could have blunted such associations. Our data expand current knowledge about BAT in people living in tropical areas and reinforce the need for taking into consideration the mean outdoor temperature before interpreting the results.

Another important outcome of this study was the demonstration of strong positive associations between the TG content of BAT with body adiposity and cardiometabolic risk markers. The strongest correlations were found between TG content and visceral adiposity (trunk fat, waist circumference, and visceral adipose tissue). Indeed, the BAT function is reduced in obesity, and a whitening of the tissue occurs. It has been shown that the “whitening” of BAT can be induced by different factors, some of them directly associated with WAT expansion, like β-adrenergic signaling impairment (55). Moreover, it has been extensively shown that the visceral adipose tissue, unlike subcutaneous adipose tissue, is implicated in insulin resistance, inflammation, and cardiovascular risk (56, 57). In concert, studies conducted in Finland showed positive associations between the Hounsfield units and waist circumference (58) and with the TG content of BAT and markers of adiposity (16). We also found positive associations between BAT TG and LDL, total cholesterol, and the Castelli indexes. Furthermore, we detected a negative association between HDL-c and the TG content of BAT. Interestingly, data from experimental studies have evidenced the role of BAT activation in reducing cardiovascular risk (8, 59, 60). It has been reported that activating thermogenic adipocytes can lead to increased HDL-c flux from plasma into feces (8) and that BAT activation increases the uptake of fatty acids from TG-rich lipoproteins into BAT (60), indicating a potential mechanism associating BAT activation and CVD risk reduction. Similar positive associations between BAT fat content and cardiometabolic risk factors were detected in Finland, as the triglyceride content of the supraclavicular area was shown to predict whole-body insulin resistance independently of BAT activity (16). In another study, it was shown that the TG content of BAT was associated with the radiodensity of adipose tissue, which in turn was strongly correlated with plasma triglycerides and the M-value, an indicator of insulin sensitivity (58); this finding was reproduced by others (17) while studying patients with glucose intolerance and diabetes. In our cohort, there were no correlations of BAT TG with fasting glucose, blood insulin, or HOMA-IR values, which could reflect the fact that normal glucose was one of the inclusion criteria. Interestingly, most of the associations were maintained after adjustment for confounding factors using logistic regression analysis. Even after adjusting for sex and age, the odds ratio of having a higher TG content increased with elevated tertiles of measures of adiposity and cardiometabolic risk factors, which could indicate the potential for this measurement to be used to differentiate distinct cardiometabolic profiles.

Another dissimilarity between this study and some others performed in temperate climate regions was the absence of association between BAT activity, volume, and TG content with CXCL14 (61), FGF21 (62), and 12-13 di-HOME (63). CXCL14 is a chemokine secreted by BAT in response to thermogenic stimuli and M2 macrophage signaling, which can increase insulin action and mitigate systemic glucose intolerance (61). FGF21 is produced in the liver in response to cold and physical activity and acts in the BAT, inducing the expression of thermogenic proteins such as PGC-1a and UCP1 (61). 12-13 di-HOME is a subproduct of unsaturated fatty acid metabolism in the BAT; once produced, 12-13 di-HOME is secreted and acts in an autocrine loop to stimulate brown adipocyte thermogenesis (63). We did not investigate the reasons for the lack of association between these markers and BAT activity in our cohort, but this could be due to the sample size, or because in participants living in the tropical areas these correlations are lost. If that is the case, it could indicate that measurements of these putative markers of BAT activity should be performed with caution in studies evaluating people not frequently exposed to environmental cold.

Because of the scarcity of data reporting BAT determinations in tropical areas and the discrepancies found in this study, we evaluated all studies performed in tropical regions and compared their findings with studies performed in temperate areas (31, 33, 38, 52, 58, 64–66). Despite the lack of standardization between studies, studies conducted between the parallels 25S and 25N indicate that there is a difference in the activation of BAT throughout the year (**Supplementary Table S4**) (24). Interestingly, some of the non-temperate-climate studies were conducted throughout the year, indicating that the seasonal effect may not be as evident in these countries. In fact, our study was carried out during autumn, winter, and spring and we detected no differences in BAT activity when comparing the outdoor temperatures of the study day and the participant’s BAT activity. On the other hand, there are not enough studies that have investigated the TG content of BAT in tropical countries, and the effects of a mild winter followed by high temperatures could have a long-term effect on BAT activation. Although it has been reported that the season can affect BAT activation, it is not known whether constant warm weather could affect BAT activity and its composition. Nevertheless, studies carried out in mice have shown that warm acclimation is able to induce the whitening of BAT (55).

This study has some limitations. The cross-sectional design precludes establishing a cause/effect relationship and the number of volunteers may have interfered with the results. On the other hand, BAT studies using PET/MRI are complex and expensive, and we were able to include 45 volunteers in the study, which could be regarded as an important strength. The number of subjects with BMI>25 might seem low for clinical purposes but is admissible for BAT PET/MRI studies (18, 19). Although our sample size calculation indicated that we had enough subjects to detect variability in BAT, whether parameters related to BAT structure and function, such as SUV, volume, and BAT TG content, would be associated with markers of CVD is not known because these comparisons have never been performed in previous studies. Therefore, we could not calculate a sample size for each analyzed variable. Nevertheless, the associations between the BAT TG and clinical markers were reinforced by the ANOVA, indicating how much the tertiles or categories of clinical markers differ from each other regarding BAT parameters. Additionally, dynamic data from our group (23) showing a lower glucose uptake rate when compared to temperate-climate countries may corroborate the current findings. The lack of an objective shivering measurement, such as electromyography, may also have affected our results. It is also important to emphasize that, although the study was performed in a tropical climate country, the indoor temperature (i.e., use of daily air-condition) could have affected the results. Nevertheless, our previous data indicate no differences in PET scans carried out throughout the different seasons (23).

In summary, in this study carried out in a non-temperate climate, we detected no associations between BAT activity, volume, or metabolic activity and markers of adiposity or cardiometabolic risk. On the other hand, we found strong and positive associations between BAT fat content and markers of adiposity, especially the visceral fat, and cardiovascular risk markers, which could be explained, at least in part, by a whitening effect of BAT in these subjects. Further studies should determine whether PET/MRI is the most appropriate method for studying BAT in tropical areas.

## Data availability statement

The raw data supporting the conclusions of this article will be made available by the authors, without undue reservation.

## Ethics statement

The studies involving human participants were reviewed and approved by Faculty of Medical Science of the University of Campinas (CAAE 60698716.1.0000.5404), Faculty of Medicine of the University of São Paulo (CAAE 60698716.1.0000.5404), and the School of Public Health of the University of São Paulo (CAAE: 60698716.1.3002.5421). The patients/participants provided their written informed consent to participate in this study.

## Author contributions

MM-P, GR-S, GAN, SRGF, and MTS performed data collection. MM-P,GR-S, PD, MU-D, GAN, MTS, SRGF, KAV, and LAV performed dataanalysis and interpretation. MM-P and LAV drafted themanuscript. LAV supervised the study. All authors edited andapproved the final manuscript.

## Funding

This study was financially supported by grants from São Paulo Research Foundation (FAPESP). Grant numbers: #2013/07607-8; #2016/10616–7; #2017/22586-8; #2018/05479-6; #2019/02055-3.

## Conflict of interest

The authors declare that the research was conducted in the absence of any commercial or financial relationships that could be construed as a potential conflict of interest.

## Publisher’s note

All claims expressed in this article are solely those of the authors and do not necessarily represent those of their affiliated organizations, or those of the publisher, the editors and the reviewers. Any product that may be evaluated in this article, or claim that may be made by its manufacturer, is not guaranteed or endorsed by the publisher.
