# Supplementary material for: Brown fat triglyceride content is associated with cardiovascular risk markers in adults from a tropical region
Source: Front Endocrinol (Lausanne). 2022 Jul 19;13:919588. doi: 10.3389/fendo.2022.919588 (PMC9343995; doi:10.3389/fendo.2022.919588)
Supplement: Supplementary file 1 [file DataSheet_1.docx]

**Supplementary file**

**Brown fat triglyceride content is associated with cardiovascular risk markers in adults from a tropical region**

Milena Monfort-Pires^1^*, Giulianna Regeni-Silva^2^, Prince Dadson^3^, Guilherme A. Nogueira^1^, Mueez U-Din^3,4^, Sandra R. G. Ferreira^5^, Marcelo Tatit Sapienza^6^, Kirsi A. Virtanen, ^3,4,7^, Licio A. Velloso^1,^*

1. Laboratory of Cell Signaling, Obesity and Comorbidities Research Center, State University of Campinas (UNICAMP), 13084-970, Campinas, São Paulo, Brazil.
2. Department of Nutrition, School of Public Health - University of São Paulo, São Paulo, SP, Brazil.
3. Turku PET Centre, University of Turku, Turku, Finland
4. Turku PET Centre, Turku University Hospital, Turku, Finland.
5. Department of Epidemiology, School of Public Health - University of São Paulo, São Paulo, SP, Brazil.
6. Division of Nuclear Medicine, Department of Radiology and Oncology, Medical School of University of São Paulo (FMUSP), São Paulo, Brazil.
7. Clinical Nutrition, Institute of Public Health and Clinical Nutrition, University of Eastern Finland (UEF), Kuopio, Finland.

**Supplementary Figure 1.** Flow chart of participants enrolled in the study


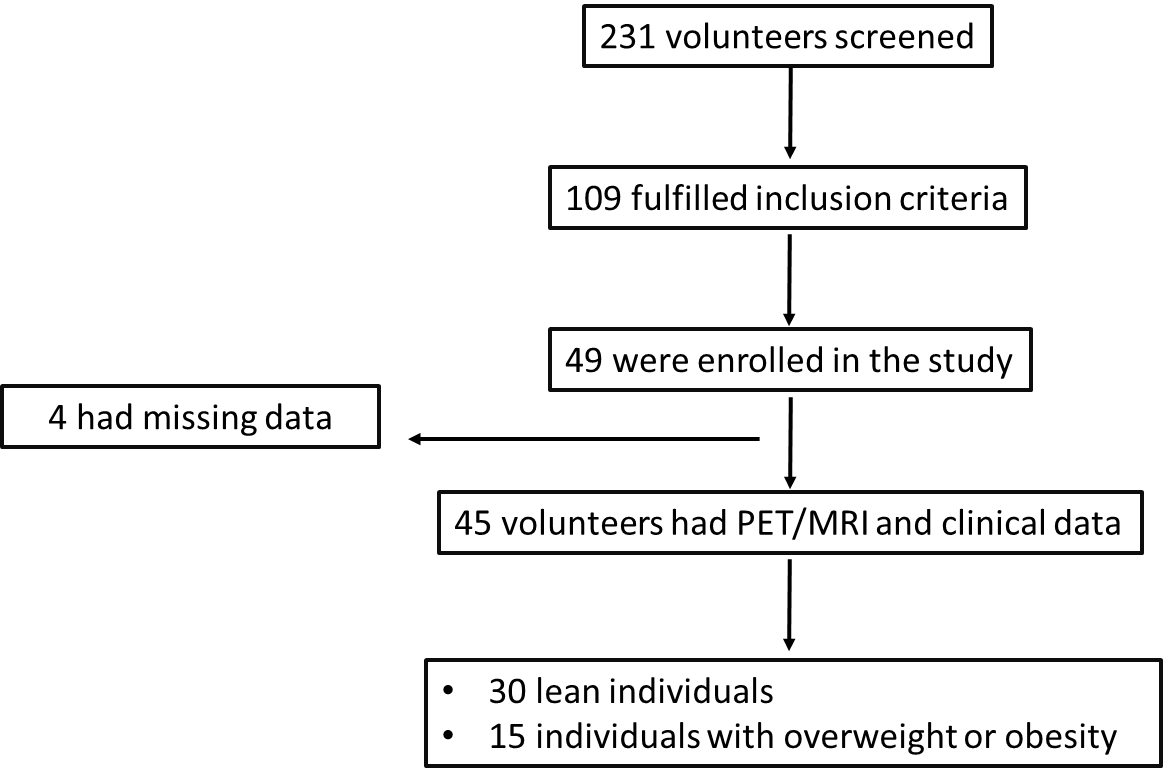


**Supplementary Figure 2. Example of visceral fat area used to calculate visceral fat mass in one volunteer with obesity (A) and one lean volunteer (B).**


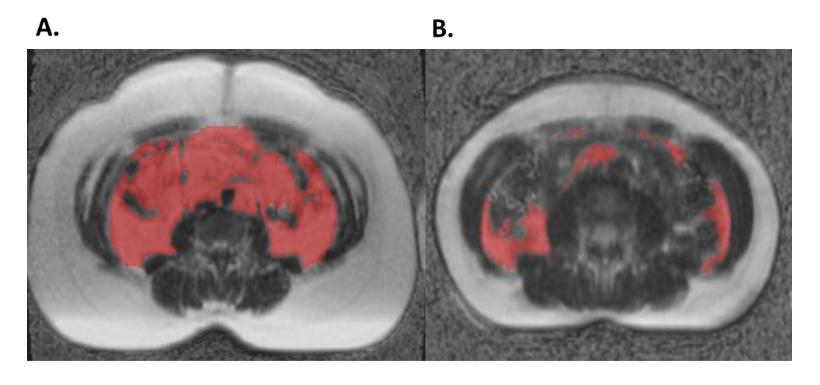


**Supplementary Table 1.** Dietary intake and physical activity data of lean participants and participants with overweight and obesity

|  | Lean  (n = 30) | Overweight/  obesity  (n = 15) | P | Total sample |
| --- | --- | --- | --- | --- |
| *Physical activity* |  |  |  |  |
| - Total physical activity (min/week) ^¥^ | 283,8±233,721 | 215,69±321,923 | 0.06 | 263,2±261,4 |
| - Leisure-time physical activity (min/week) ^¥^ | 183,17±235,138 | 170±275,862 | 0.49 | 179,2±244,9 |
| - Seating time (hours)^¥^ | 46,9±17.0 | 40.2 ±14.6 | 0.20 | 44,9±16,4 |
| *Dietary data* |  |  |  |  |
| - Energy intake (kcal) | 2012,4±507,5 | 2104,1±611,6 | 0.60 | 2042,9±539,2 |
| - Carbohydrates (% of total energy intake) | 47,3±10,3 | 45,4±8,6 | 0.53 | 46,7±9,7 |
| - Fat (% of total energy intake) | 34,1±7,2 | 35,9±6 | 0.40 | 34,7±6,8 |
| - Protein (% of total energy intake) | 16,9±4,7 | 18,4±5,1 | 0.33 | 1,2±3 |

**Supplementary Table 2. Spearman’s correlations between BAT activity, SUV max, volume, metabolic activity, and content of TG in the total sample.**

|  | BAT activity (mean SUV) | BAT SUV max | BAT volume (mL) | BAT metabolic activity (SUV*mL) | BAT content of TG (%) |
| --- | --- | --- | --- | --- | --- |
| BAT activity (mean SUV) | 1 | .914** | .863** | .888** | -0.186 |
| BAT SUV max | .914** | 1 | .899** | .911** | -0.065 |
| BAT volume (mL) | .863** | .899** | 1 | .996** | 0.01 |
| BAT metabolic activity (SUV*mL) | .888** | .911** | .996** | 1 | -0.01 |
| BAT content of TG (%) | -0.186 | -0.065 | 0.01 | -0.01 | 1 |

- P<0.05 / ** p<0.01

**
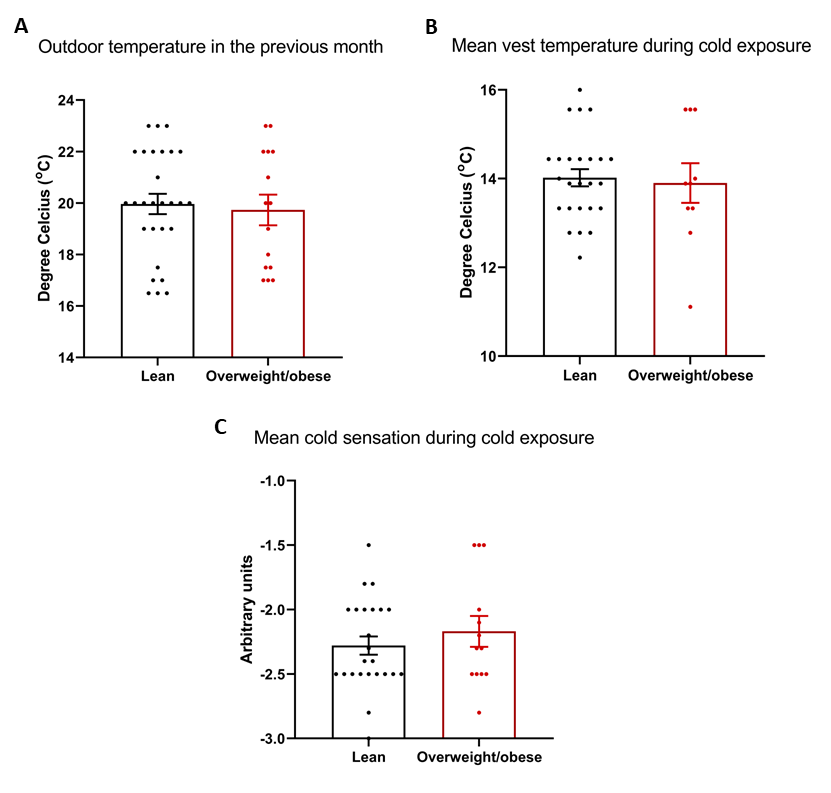
Supplementary Figure 3. Mean outside temperature, mean vest temperature, and col sensation in lean volunteers and volunteers with overweight and obesity.** The outdoor temperature in the previous month (A), mean vest temperature during cold exposure (B) and cold sensation (C).

**Supplementary Figure 4. Heatmap of correlations between brown adipose tissue with body adiposity and circulating biomarkers.** BAT activity (mean SUV), BAT maximum SUV, BAT volume (mL), BAT metabolic activity (SUV*mL) and BAT content of triglycerides (A), and biomarkers (B) with age and body composition parameters in lean volunteers. Different colors indicate the intensity of the correlation coefficient (from the darkest red with correlation coefficient of -1.0 to the darkest blue with correlation coefficient of 1.0)

**
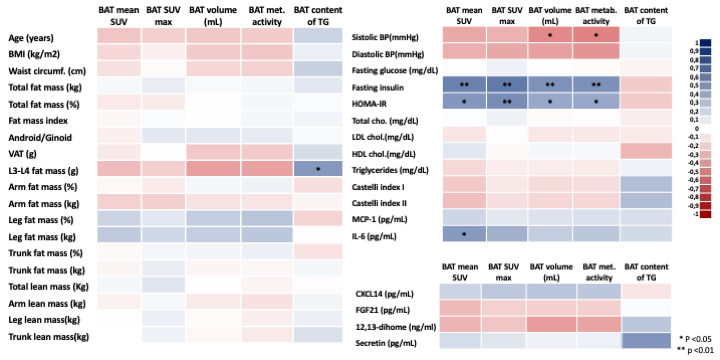
**

**Supplementary Figure 5. Heatmap of correlations between brown adipose tissue with body adiposity and circulating biomarkers.** BAT activity (mean SUV), BAT maximum SUV, BAT volume (mL), BAT metabolic activity (SUV*mL) and BAT content of triglycerides (A), and biomarkers (B) with age and body composition parameters in volunteers with overweight and obesity. Different colors indicate the intensity of the correlation coefficient (from the darkest red with correlation coefficient of -1.0 to the darkest blue with correlation coefficient of 1.0)

**
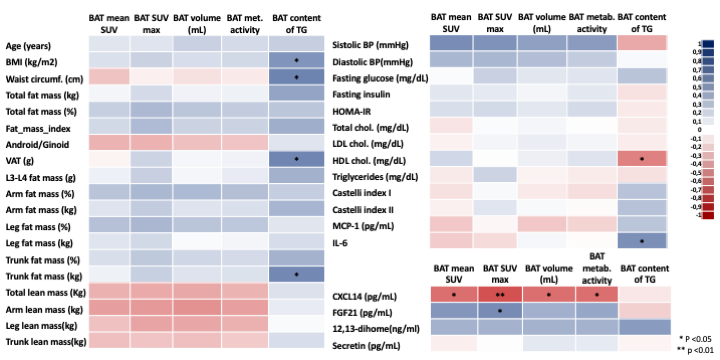
**

**Supplementary Table 3. Univariate and multivariate logistic regression analyses of BAT activity, volume, and content of triglycerides with body composition variables and markers of cardiovascular risk in the total sample. Clinical variables were analyzed according to categories (BMI and waist circumference) or as tertiles.**

|  | Univariate analysis | | | | |  | Multivariate analysis* | | | | |  |
| --- | --- | --- | --- | --- | --- | --- | --- | --- | --- | --- | --- | --- |
|  | **BAT SUV (<1.2/≥1.2)** | **p** | **BAT volume (no/yes)** | **p** | **BAT content of TG (<72/≥72%)** | **p** | **BAT SUV (<1.2/≥1.2)** | **p** | **BAT volume (no/yes)** | **p** | **BAT content of TG (<72/≥72%)** | **p** |
| Sex | |  |  |  |  |  |  |  |  |  |  |  |
| Male | 1.0 (reference) |  | 1.0 (reference) |  | 1.0 (reference) |  | 1.0 (reference) |  | 1.0 (reference)** |  | 1.0 (reference) |  |
| Female | 4.500 (0.844 - 23.981) | 0.078 | 1.750 (0.471 - 6.502) | 0.40 | 0.346 (0.095 - 1.267) | 0.11 | 5.244 (0.936 - 29.370) | 0.059 | 1.788 (0.476 - 6.717) | 0.39 | 0.244 (0.057 - 1.040) | 0.06 |
| Age |  |  |  |  |  |  |  |  |  |  |  |  |
| <30 | 1.0 (reference) |  | 1.0 (reference) |  | 1.0 (reference) |  | 1.0 (reference) |  | 1.0 (reference)*** |  | 1.0 (reference) |  |
| ≥30 | 0.347 (0.086 - 1.404) | 0.14 | 0.667 (0.167 - 2.666) | 0.57 | 2.880 (0.747 - 11.096) | 0.12 | 0.918 (0.789 - 1.068) | 0.27 | 0.982 (0.856 - 1.125) | 0.79 | 1.134 (0.980 - 1.311) | 0.09 |
| BMI |  |  |  |  |  |  |  |  |  |  |  |  |
| <24.99 | 1.0 (reference) |  | 1.0 (reference) |  | 1.0 (reference) |  | 1.0 (reference) |  | 1.0 (reference) |  | 1.0 (reference) |  |
| >25 | 1.195 (0.288 - 4.959) | 0.81 | 4.206 (0.791 - 22.359) |  | 36.000 (39.55 - 327.716) | <0.01 | 1.721 (0.356 - 8.311) | 0.50 | 5.006 (0.876 - 28.609) | 0.07 | 35.601 (3.807 - 332.881) | <0.01 |
| Waist circumference (cm) | |  |  |  |  |  |  |  |  |  |  |  |
| <80(F)/<90 (M) | 1.0 (reference) |  | 1.0 (reference) |  | 1.0 (reference) |  | 1.0 (reference) |  | 1.0 (reference) |  | 1.0 (reference) |  |
| ≥80(F)/ ≥90 (M) | 1.528 (0.385 - 6.070) | 0.55 | 2.917 (0.666 - 12.764) | 0.16 | 21.250 (3.713 - 121.606) | 0.01 | 1.835 (0.432 - 7.798) | 0.41 | 3.347 (0.705 - 15.893) | 0.128 | 27.055 (3.722 - 196.651) | <0.01 |
| Total Fat mass (kg) (tertiles) | |  |  |  |  |  |  |  |  |  |  |  |
| 1st | 1.0 (reference) |  | 1.0 (reference) |  | 1.0 (reference) |  | 1.0 (reference) |  | 1.0 (reference) |  | 1.0 (reference) |  |
| 2nd | 1.576 (0.288 - 8.614) | 0.60 | 2.750 (0.583 - 12.975) | 0.20 | 1.333 (0.242 - 7.348) | 0.74 | 1.446 (0.237 8.842) | 0.69 | 2.553 (0.529 - 12.333) | 0.24 | 4.578 (0.447 - 46.832) | 0.20 |
| 3rd | 1.733 (0.314 - 9.573) | 0.53 | 6.000 (0.965 - 37.296) | 0.055 | 34.667 (3.057 - 393.185) | <0.01 | 2.196 (0.346 - 13.961) | 0.40 | 6.27 (0.970 - 40.524) | 0.054 | 184.200 (6.236 - 5,440.93) | <0.01 |
| Total Fat mass (%) (tertiles) | |  |  |  |  |  |  |  |  |  |  |  |
| 1st | 1.0 (reference) |  | 1.0 (reference) |  | 1.0 (reference) |  | 1.0 (reference) |  | 1.0 (reference) |  | 1.0 (reference) |  |
| 2nd | 1.576 (0.288 - 8.614) | 0.60 | 1.200 (0.267 - 5.400) | 0.81 | 1.667 (0.333 - 9.157) | 0.56 | 1.660 (0.269 - 10.234) | 0.59 | 1.193 (0.260 - 5.482) | 0.82 | 1.766 (0.219 - 14.223) | 0.59 |
| 3rd | 1.733 (0.314 - 9.573) | 0.53 | 4.000 (0.649 - 24.660) | 0.14 | 39.00 (3.477 - 437.49) | <0.01 | 2.182 (0.350 - 13.614) | 0.40 | 4.036 (0.646 - 25.210) | 0.14 | 101.823 (5.416 - 1,914.364) | <0.01 |
| Visceral adipose tissue (tertiles) | | |  |  |  |  |  |  |  |  |  |  |
| 1st | 1.0 (reference) |  | 1.0 (reference) |  | 1.0 (reference) |  | 1.0 (reference) |  | 1.0 (reference) |  | 1.0 (reference) |  |
| 2nd | 0.750 (0.137 - 4.095) | 0.74 | 2.750 (0.583 - 12.976) | 0.20 | 3.750 (0.589 - 23.867) | 0.16 | 0.639 (0.104 - 3.934) | 0.63 | 1.970 (0.406 - 9.558) | 0.40 | 9.057 (0.862 - 95.147) | 0.07 |
| 3rd | 1.200 (0.237 - 6.065) | 0.83 | 6.000 (0.965 - 37.296) | 0.055 | 65.00 (5.136 - 822.593) | <0.01 | 1.669 (0.274 - 10.181) | 0.88 | 3.047 (0.532 - 17.442) | 0.21 | 189.070 (8.280 -4,317.164) | <0.01 |
| Trunk percentage of fat (tertiles) | |  | |  |  |  |  |  |  |  |  |  |
| 1st | 1.0 (reference) |  | 1.0 (reference) |  | 1.0 (reference) |  | 1.0 (reference) |  | 1.0 (reference) |  | 1.0 (reference) |  |
| 2nd | 1.576 (0.288 - 8.614) | 0.60 | 1.667 (0.353 - 7.875) | 0.52 | 0.800 (0.151 - 4.245) | 0.79 | 1.274 (0.203 - 7.990) | 0.80 | 1;552 (0.303 - 7.947) | 0.60 | 1.439 (0.196 - 10.551) | 0.72 |
| 3rd | 1.733 (0.314 - 9.573) | 0.53 | 2.444 (0.473 - 12.629) | 0.29 | 26.00 (2.451 - 275.826) | <0.01 | 2.019 (0.328 - 12.424) | 0.45 | 2.436 (0.465 - 12.758) | 0.29 | 72.788 (4.115 - 1287.456) | 0.03 |
| HDL cholesterol | |  |  |  |  |  |  |  |  |  |  |  |
| 1st | 1.0 (reference) |  | 1.0 (reference) |  | 1.0 (reference) |  | 1.0 (reference) |  | 1.0 (reference) |  | 1.0 (reference) |  |
| 2nd | 4.875 (0.785 – 20.289) | 0.09 | 1.667 (0.311 – 8.928) | 0.55 | 0.051 (0.007 – 0.372) | <0.01 | 3.052 (0.399 – 23.333) | 0.28 | 1.434 (0.213 – 9.628) | 0.71 | 0.053 (0.006 – 0.474) | <0.01 |
| 3rd | 1.083 (0.131 – 8.946) | 0.94 | 1.125 (0.229 -5.537) | 0.89 | 0.088 (0.013 -0.606) | <0.01 | 0.70 (0.073 – 6.906) | 0.94 | 1.032 (0.186 – 5.712) | 0.97 | 0.088 (0.011 – 0.699) | 0.02 |
| Castelli Index I | |  |  |  |  |  |  |  |  |  |  |  |
| 1st | 1.0 (reference) |  | 1.0 (reference) |  | 1.0 (reference) |  | 1.0 (reference) |  | 1.0 (reference) |  | 1.0 (reference) |  |
| 2nd | 0.750 (0.135 – 4.165) | 0.74 | 0.436 (0.080 – 2.380) | 0.34 | 2.143 (0.376 – 12.197) | 0.39 | 1.210 (0.184 – 7.965) | 0.84 | 0.502 (0.085 – 2.945) | 0.45 | 1.218 (0.161 – 9.233) | 0.85 |
| 3rd | 0.750 (0.135 – 4.165) | 0.74 | 0.682 (0.122 – 3.825) | 0.66 | 18.00 (2.468 – 131.285) | <0.01 | 1.609 (0.226 – 11.453) | 0.64 | 0.800 (0.120 – 5.339) | 0.82 | 13.735 (1.552 – 121.542) | 0.02 |
| Castelli Index II | |  |  |  |  |  |  |  |  |  |  |  |
| 1st | 1.0 (reference) |  | 1.0 (reference) |  | 1.0 (reference) |  | 1.0 (reference) |  | 1.0 (reference) |  | 1.0 (reference) |  |
| 2nd | 0.825 (0.147 – 4.628) | 0.83 | 0.545 (0.095 – 3.146) | 0.50 | 1.905 (0.330 – 11.009) | 0.47 | 1.324 (0.197 – 8.892) | 0.77 | 0.601 (0.097 – 3.719) | 0.60 | 1.305 (0.180 – 9.478) | 0.54 |
| 3rd | 0.688 (0.125 – 3.786) | 0.67 | 0.545 (0.103 – 2.892) | 0.48 | 10.667 (1.705 – 66.720) | 0.01 | 1.612 (0.222 – 11.696) | 0.64 | 0.625 (0.095 – 4.113) | 0.63 | 7.518 (0.955 – 59.195) | 0.055 |
| Interleukin-6 | |  |  |  |  |  |  |  |  |  |  |  |
| 1st | 1.0 (reference) |  | 1.0 (reference) |  | 1.0 (reference) |  | 1.0 (reference) |  | 1.0 (reference) |  | 1.0 (reference) |  |
| 2nd | . | 1.0 | 1.667 (0.286 – 9.708) | 0.57 | 0.250 (0.043 – 1.443) | 0.12 | . | 1.0 | 1.547 (0.258 – 9.270) | 0.63 | 0.220 (0.033 – 1.452) | 0.12 |
| 3rd | . | 1.0 | 1.00 (0.200 – 5.004) | 1.0 | 4.583 (0.551 – 29.527) | 0.12 | . | 1.0 | 0.983 (0.179 – 5.397) | 0.98 | 3.734 (0.462 – 30.198) | 0.22 |
| MCP-1 | |  |  |  |  |  |  |  |  |  |  |  |
| 1st | 1.0 (reference) |  | 1.0 (reference) |  | 1.0 (reference) |  | 1.0 (reference) |  | 1.0 (reference) |  | 1.0 (reference) |  |
| 2nd | 0.161 (0.015 – 1.678) | 0.13 | 0.333 (0.062 – 1.779) | 0.20 | 1.400 (0.296 – 6.662) | 0.67 | 0.175 (0.014 – 2.196) | 0.18 | 0.399 (0.069 – 2.311) | 0.31 | 1.070 (0.185 – 6.208) | 0.94 |
| 3rd | 0.900 (0.172 – 4.699) | 0.90 | 2.000 (0.274 – 14.587) | 0.49 | 3.733 (0.646 – 21.557) | 0.14 | 2.099 (0.279 – 15.798) | 0.47 | 2.509 (0.321 – 19.606) | 0.38 | 2.561 (0.402 – 16.337) | 0.32 |

* Multivariate analysis: adjusted for age and sex /** Adjusted for age/***Adjusted for sex

**Supplementary Table 4. Summary of studies showing BAT activity (in mean or max SUV) in tropical and temperate climate countries**

| **Manuscript title** | **Country** | **Population** | **Age** | **City** | **Type of PET/CT** | **Measurement** | **SUV** | **Reference** |
| --- | --- | --- | --- | --- | --- | --- | --- | --- |
| Brown adipose tissue activity is reduced in women with polycystic ovary syndrome | Brazil | 70 women (Polycystic ovary syndrome) | 18-45 years | Belo Horizonte | Static | SUV max | SUV max median 7.4 g/mL (0.9-15.4) and 13 g/mL (4.7-18.4) | Oliveira et al, 2019 [1] |
| Brown Adipose Tissue: Multimodality Evaluation by PET, MRI, Infrared Thermography, and Whole‐Body Calorimetry (TACTICAL‐II) | Singapore | 20 men/women | Mean age 26 | . | Static | SUV (mean) | SUV mean 1-1.8 g/mL (mean 1.51) | Sun et al, 2019 [2] |
| Chronic ephedrine administration decreases brown adipose tissue activity in a randomized controlled human trial: implications for obesity | Australia | 23 men | Mean age 23 years | Melbourne | Static | SUV max/ SUV mean (ephedrine stimulation) | SUV max 0.98/0.96 g/mL and 0.7 g/mL SUV mean | Carey et al, 2015 [3] |
| Distinct regulation of hypothalamic and brown/beige adipose tissue activities in human obesity | Brazil | 12 lean/12 obese | Age range 18-45 years | Campinas | Static | SUV max | 0-12 g/mL | Rachid et al, 2015 [4] |
| Effect of pioglitazone treatment on brown adipose tissue volume and activity and hypothalamic gliosis in patients with type 2 diabetes mellitus: a proof-of-concept study | Brazil | 6 men/women (80%) | Mean age 47 years | Campinas | Static | Mean SUV/body mass | 0.34-.0.36 g/mL | De Lima-Junior et al, 2019 [5] |
| Effects of Short-Term Metformin Treatment on Brown Adipose Tissue Activity and Plasma Irisin Levels in Women with Polycystic Ovary Syndrome: A Randomized Controlled Trial | Brazil | 45 women (Polycystic ovary syndrome) | Mean age 31 years | Belo Horizonte | Static | SUV max/body lean mass | SUV max 7.4/5.2 g/mL | Oliveira et al, 2020 [6] |
| Ephedrine activates brown adipose tissue in lean but not obese humans | Australia | 9 lean/9 obese | Mean age 25/27 years | Melbourne | Static | SUV max/ephedrine | 2.1 g/mL and 1.0 g/mL (after ephedrine) | Carey et al, 2013 [7] |
| Glucocorticoids suppress brown adipose tissue function in humans: A double-blind placebo-controlled study | Australia | 13 (6 men/7 women) | Mean age 28 years | Brisbane | Static | SUV max | SUV max 6.1/3.7 g/mL | Thuzar et al, 2018 [8] |
| Impairment of body mass reduction-associated activation of brown/beige adipose tissue in patients with type 2 diabetes mellitus | Brazil | 39 (11 lean, 14 obese non-diabetic, 14 obese and diabetic) | Mean age 35.6 years | Campinas | Static | SUV max | 1.56 / 3.59 g/mL (lean)/1.02 g/mL (Obese Non-Diabetic)/1.35g/mL (Obese Diabetic) | Rodovalho et al 2017 [9] |
| Mineralocorticoid antagonism enhances brown adipose tissue function in humans: A randomized placebo-controlled cross-over study | Australia | 10 (8 women/2 men) | Age range 18-40 years | Brisbane | Static | SUV max | SUV max 6.3/3.98 (g/mL) | Thuzar et al, 2019 [10] |
| Short dietary intervention with olive oil increases brown adipose tissue activity in lean but not overweight individuals | Brazil | 41 (29 lean, 15 overweight) | Age range 25-40 years | São Paulo | Static/dynamic | SUV mean, SUV max | mean SUV 0.8/0.6 g/mL, SUV max 1.8-1.9 /GUR: 0.94-1.0 µmol × min-1 × 100 g-1 | Monfort-Pires et al, 2021[11] |
| Sleep duration and quality are not associated with brown adipose tissue volume or activity-as determined by 18F-FDG uptake, in young, sedentary adults | Spain | 118 (69% women) | Mean age 22 years | Granada | Static | SUV mean/SUV peak | SUV mean 3.74 g/mL, peak 11.19 g/mL | Acosta et al, 2019 [12] |
| Brown Adipose Tissue Activation Is Linked to Distinct Systemic Effects on Lipid Metabolism in Humans | USA | 16 men | Mean age 48 years | Galveston | Static | Mean SUV | Mean SUV 1.9 g/mL | Chondronikola et al, 2016 [13] |
| Brown Adipose Tissue Improves Whole-Body Glucose Homeostasis and Insulin Sensitivity in Humans | USA | 12 men (overweight/obese) | Age range 41-49 years | Galveston | Static | Mean SUV | Mean SUV 1.6 (-) 2.2 (+) g/mL | Chondronikola et al, 2014 [14] |
| Brown adipose tissue oxidative metabolism contributes to energy expenditure during acute cold exposure in humans | Canada | 6 men | Age range 23-42 years | Quebec | Dynamic | Glucose uptake rate | 10.8 ± 4.5 μmol/min (range 1.3 to 28.7 μmol/min) | Ouellet et al, 2012 [15] |
| High Brown Fat Activity Correlates with Cardiovascular Risk Factor Levels Cross-Sectionally and Subclinical Atherosclerosis at 5-Year Follow-Up | Finland | 31(6 men) | Mean age 41 years | Turku | Dynamic | Glucose uptake rate | Median GUR 2.40 μmoL/100 g per minute | Raiko et al, 2020 [16] |
| Metabolically Active Brown Adipose Tissue Is Found in Adult Subjects with Type 1 Diabetes | Sweden | 11 type 1 diabetes mellitus subjects | Age range 29-32 years | Upsala | Dynamic | Glucose uptake rate | Mean GUR 0.75-38.7 µmol × min-1 × 100 g-1 | Eriksson et al, 2019 [17] |
| Human Brown Adipose Tissue Temperature and Fat Fraction Are Related to Its Metabolic Activity | Finland | 10 men/women | Age range 25-45 years | Turku | Dynamic | Glucose uptake rate | GUR 15.7 ± 8.9 µmol/100 g/min | Koskensalo et al, 2017 [18] |
| Blunted metabolic responses to cold and insulin stimulation in brown adipose tissue of obese humans | Finland | 36 obese/27 lean | Mean age 38-39 years | Turku | Dynamic | Glucose uptake rate | GUR 3-6 µmol/100 g/min | Orava et al 2013[19], |
| Different Metabolic Responses of Human Brown Adipose Tissue to Activation by Cold and Insulin | Finland | 27 men/women | Mean age 38-41 years | Turku | Dynamic | Glucose uptake rate | GUR 9.1 µmol/100 g/min | Orava et al, 2011 [20] |
| Functional Brown Adipose Tissue in Healthy Adults | Finland | 5 men/women | Age range 20-50 years | Turku | Dynamic | Glucose uptake rate | GUR 5-17 µmol/100 g/min | Virtanen et al, 2009 [21] |
| Cold-Activated Brown Adipose Tissue is Associated with Less Cardiometabolic Dysfunction in Young Adults with Obesity | USA | 44 (21 women/23 men) | Mean age 23.6-26.4 years | Salt Lake City | Static | Mean SUV | Mean SUV 3.6-4.3 g/ml | Mihalopoulos et al, 2020 [22] |
| Sexual Dimorphisms in Adult Human Brown Adipose Tissue | USA | 24 (12 men/12 women) | Age range 18-35 years | Bethesda | Static | Mean SUV | mean SUV 4.91-5.01 g/mL | Fletcher et al, 2020 [23] |
| Repeatability of Quantitative Brown Adipose Tissue Imaging Metrics on Positron Emission Tomography with 18F-Fluorodeoxyglucose in Humans | USA | 24 subjects | Mean age 19.3-31.9 years | Washington | Static | SUV max | SUV max 7.6-9.2 g/mL | Fraum et al, 2019 [24] |
| Increased Brown Adipose Tissue Oxidative Capacity in Cold-Acclimated Humans | Canada | 6 subjects | Mean age 23 years | Ottawa | Dynamic | NET glucose uptake | NET glucose uptake 163 nmol·g−1·min−1 | Blondin et al, 2014[25] |

1. Oliveira, F.R., et al., *Brown adipose tissue activity is reduced in women with polycystic ovary syndrome.* Eur J Endocrinol, 2019. **181**(5): p. 473-480.DOI: 10.1530/EJE-19-0505.

2. Sun, L., et al., *Brown Adipose Tissue: Multimodality Evaluation by PET, MRI, Infrared Thermography, and Whole-Body Calorimetry (TACTICAL-II).* Obesity (Silver Spring), 2019. **27**(9): p. 1434-1442.DOI: 10.1002/oby.22560.

3. Carey, A.L., et al., *Chronic ephedrine administration decreases brown adipose tissue activity in a randomised controlled human trial: implications for obesity.* Diabetologia, 2015. **58**(5): p. 1045-54.DOI: 10.1007/s00125-015-3543-6.

4. Rachid, B., et al., *Distinct regulation of hypothalamic and brown/beige adipose tissue activities in human obesity.* Int J Obes (Lond), 2015. **39**(10): p. 1515-22.DOI: 10.1038/ijo.2015.94.

5. de-Lima-Junior, J.C., et al., *Effect of pioglitazone treatment on brown adipose tissue volume and activity and hypothalamic gliosis in patients with type 2 diabetes mellitus: a proof-of-concept study.* Acta Diabetol, 2019. **56**(12): p. 1333-1339.DOI: 10.1007/s00592-019-01418-2.

6. Oliveira, F.R., et al., *Effects of Short Term Metformin Treatment on Brown Adipose Tissue Activity and Plasma Irisin Levels in Women with Polycystic Ovary Syndrome: A Randomized Controlled Trial.* Horm Metab Res, 2020. **52**(10): p. 718-723.DOI: 10.1055/a-1157-0615.

7. Carey, A.L. and B.A. Kingwell, *Brown adipose tissue in humans: therapeutic potential to combat obesity.* Pharmacol Ther, 2013. **140**(1): p. 26-33.DOI: 10.1016/j.pharmthera.2013.05.009.

8. Thuzar, M., et al., *Glucocorticoids suppress brown adipose tissue function in humans: A double-blind placebo-controlled study.* Diabetes Obes Metab, 2018. **20**(4): p. 840-848.DOI: 10.1111/dom.13157.

9. Rodovalho, S., et al., *Impairment of body mass reduction-associated activation of brown/beige adipose tissue in patients with type 2 diabetes mellitus.* Int J Obes (Lond), 2017. **41**(11): p. 1662-1668.DOI: 10.1038/ijo.2017.152.

10. Thuzar, M., et al., *Mineralocorticoid antagonism enhances brown adipose tissue function in humans: A randomized placebo-controlled cross-over study.* Diabetes Obes Metab, 2019. **21**(3): p. 509-516.DOI: 10.1111/dom.13539.

11. Monfort-Pires, M., et al., *Short Dietary Intervention with Olive Oil Increases Brown Adipose Tissue Activity in Lean but not Overweight Subjects.* J Clin Endocrinol Metab, 2021. **106**(2): p. 472-484.DOI: 10.1210/clinem/dgaa824.

12. Acosta, F.M., et al., *Sleep duration and quality are not associated with brown adipose tissue volume or activity-as determined by 18F-FDG uptake, in young, sedentary adults.* Sleep, 2019. **42**(12).DOI: 10.1093/sleep/zsz177.

13. Chondronikola, M., et al., *Brown Adipose Tissue Activation Is Linked to Distinct Systemic Effects on Lipid Metabolism in Humans.* Cell Metab, 2016. **23**(6): p. 1200-1206.DOI: 10.1016/j.cmet.2016.04.029.

14. Chondronikola, M., et al., *Brown adipose tissue improves whole-body glucose homeostasis and insulin sensitivity in humans.* Diabetes, 2014. **63**(12): p. 4089-99.DOI: 10.2337/db14-0746.

15. Ouellet, V., et al., *Brown adipose tissue oxidative metabolism contributes to energy expenditure during acute cold exposure in humans.* J Clin Invest, 2012. **122**(2): p. 545-52.DOI: 10.1172/JCI60433.

16. Raiko, J., et al., *High Brown Fat Activity Correlates With Cardiovascular Risk Factor Levels Cross-Sectionally and Subclinical Atherosclerosis at 5-Year Follow-Up.* Arterioscler Thromb Vasc Biol, 2020. **40**(5): p. 1289-1295.DOI: 10.1161/ATVBAHA.119.313806.

17. Eriksson, O., et al., *Metabolically Active Brown Adipose Tissue Is Found in Adult Subjects with Type 1 Diabetes.* Int J Mol Sci, 2019. **20**(23).DOI: 10.3390/ijms20235827.

18. Koskensalo, K., et al., *Human Brown Adipose Tissue Temperature and Fat Fraction Are Related to Its Metabolic Activity.* J Clin Endocrinol Metab, 2017. **102**(4): p. 1200-1207.DOI: 10.1210/jc.2016-3086.

19. Orava, J., et al., *Blunted metabolic responses to cold and insulin stimulation in brown adipose tissue of obese humans.* Obesity (Silver Spring), 2013. **21**(11): p. 2279-87.DOI: 10.1002/oby.20456.

20. Orava, J., et al., *Different metabolic responses of human brown adipose tissue to activation by cold and insulin.* Cell Metab, 2011. **14**(2): p. 272-9.DOI: 10.1016/j.cmet.2011.06.012.

21. Virtanen, K.A., et al., *Functional brown adipose tissue in healthy adults.* N Engl J Med, 2009. **360**(15): p. 1518-25.DOI: 10.1056/NEJMoa0808949.

22. Mihalopoulos, N.L., et al., *Cold-Activated Brown Adipose Tissue is Associated with Less Cardiometabolic Dysfunction in Young Adults with Obesity.* Obesity (Silver Spring), 2020. **28**(5): p. 916-923.DOI: 10.1002/oby.22767.

23. Fletcher, L.A., et al., *Sexual Dimorphisms in Adult Human Brown Adipose Tissue.* Obesity (Silver Spring), 2020. **28**(2): p. 241-246.DOI: 10.1002/oby.22698.

24. Fraum, T.J., et al., *Repeatability of Quantitative Brown Adipose Tissue Imaging Metrics on Positron Emission Tomography with (18)F-Fluorodeoxyglucose in Humans.* Cell Metab, 2019. **30**(1): p. 212-224 e4.DOI: 10.1016/j.cmet.2019.05.019.

25. Blondin, D.P., et al., *Increased brown adipose tissue oxidative capacity in cold-acclimated humans.* J Clin Endocrinol Metab, 2014. **99**(3): p. E438-46.DOI: 10.1210/jc.2013-3901.
